# Exploring prognostic factors on vascular outcomes among maintenance dialysis patients and establishing a prognosis prediction model using machine learning methods

**DOI:** 10.1186/s12911-025-03302-2

**Published:** 2025-12-05

**Authors:** Chung-Kuan Wu, Zih-Kai Kao, Vy-Khanh Nguyen, Noi Yar, Ming-Tsang Chuang, Tzu-Hao Chang

**Affiliations:** 1 Division of Nephrology, Department of Internal Medicine, Shin-Kong Wu Ho-Su Memorial Hospital, Taipei, Taiwan; 2 Division of Digital Informatics Management, Department of Digital Medicine, Shin Kong Wu Ho-Su Memorial Hospital, Taipei, Taiwan; 3 School of Medicine, Fu-Jen Catholic University, New Taipei City, Taiwan; 4 Institute of Biophotonics, National Yang Ming Chiao Tung University, Taipei, Taiwan; 5 College of Management, School of Health Care Administration, Taipei Medical University, Taipei, Taiwan; 6 College of Medical Science, School of Pharmacy, University of South Australia, Adelaide, Australia; 7 Clinical Data Center, Office of Data Science, Taipei Medical University, Taipei, Taiwan; 8 Graduate Institute of Biomedical Informatics, Taipei Medical University, Taipei, Taiwan

**Keywords:** Hemodialysis, Cardiovascular disease, Major adverse cardiovascular event, Machine learning, Elastic-net

## Abstract

**Background:**

Cardiovascular diseases (CVDs) remain the leading cause of morbidity and mortality in end-stage kidney disease patients, with persistently high rates of major adverse cardiovascular events (MACEs). Traditional risk factors such as diabetes and hypertension have limited predictive value in this population, while chronic kidney disease (CKD)-specific factors including inflammation, disordered mineral metabolism, and vascular calcification play significant roles. Therefore, we developed a machine learning-based model incorporating traditional and CKD-specific variables to improve MACEs risk predictions and facilitate early intervention.

**Methods:**

We retrospectively enrolled 412 adults undergoing maintenance hemodialysis (MHD) at a single center between October and December 2018, with follow-up until December 2021 or censoring. Enrolled patients were classified by MACEs occurrences. An elastic-net regularized Cox regression with backward selection was used to identify key MACE predictors, integrating traditional and CKD-specific risk factors. The model performance was validated via leave-one-out cross-validation and area under the receiver operating characteristic curve (AUROC). Standard statistical tests were applied for group comparisons.

**Results:**

The elastic-net regression identified 46 key predictors from a high-dimensional dataset. Patients who developed MACEs were older and had higher prevalences of diabetes, hypertension, coronary disease, heart failure, and lower albumin/cholesterol. A Cox regression with backward selection was used to refine the model to 13 predictors. The final model demonstrated excellent predictive performance, (AUROC = 0.864) (95% CI, 0.8131– 0.9148), effectively stratifying patients into high- and low-risk groups with significant survival differences (log-rank *p* < 0.001).

**Conclusions:**

The machine learning-based model demonstrated high predictive accuracy for MACEs in MHD patients by integrating both traditional and CKD-specific risk factors, offering a potential tool for early identification and clinical decision-making.

**Clinical trial number:**

Not applicable.

**Supplementary Information:**

The online version contains supplementary material available at 10.1186/s12911-025-03302-2.

## Introduction

Cardiovascular diseases (CVDs) are the preeminent cause of morbidity and mortality among patients with end-stage kidney disease (ESKD) on maintenance hemodialysis (MHD) [1]. As expected, rates of major adverse cardiovascular events (MACEs) in MHD populations are particularly high. Data from various observational cohorts revealed up to 19.4 events per 100 patient-years, highlighting a global health burden that persists despite medical advancements [2]. While traditional atherogenic risk factors such as diabetes mellitus, hypertension, and dyslipidemia are highly prevalent within this population, their predictive utility is often attenuated, and thus they might fail to fully account for observed event rates [3]. Furthermore, chronic kidney disease (CKD) -specific risk factors including systemic inflammation, oxidative stress, profound mineral and bone metabolism disorders (MBD), and hemodynamic stress are critical when developing risk-stratification models for application to MHD populations [4]. Most importantly, the combination of these different risk factors and CKD-specific disturbances could extensively exacerbate vascular calcification, which is regarded as a risk factor for MACEs. For example, aortic arch calcification (AoAC) serves as a surrogate for this systemic burden of vascular diseases and is an independent predictor of cardiovascular and all-cause mortality in MHD patients [5, 6]. The progression of MACEs in MHD populations is rather complex and a result of interconnection between systemic insults. A robust predictive model therefore should integrate markers that reflect these diverse and interacting pathophysiological factors.

Malnutrition-inflammation-atherosclerosis (MIA) syndrome was identified in numerous studies as a consistent predictor of adverse outcomes in patients undergoing MHD [7–9]. Hypoalbuminemia is an indicator of MIA syndrome, reflecting the state of heightened inflammation and compromised vascular integrity, thereby making serum albumin an ideal choice for analysis within a sophisticated modeling framework [5]. Additionally, studies demonstrated that patients with persistently low hemoglobin (Hb) levels faced the highest MACE risk [10, 11]. Beyond anemia, the uremic milieu also fosters a paradoxical hemostatic defect characterized by dysfunctional platelets coexisting with a prothrombotic state [12], in which heightened platelet reactivity is likely associated with increased risks of atherothrombotic events and MACEs even under antiplatelet therapy [13]. Furthermore, previous studies suggested obvious accumulating risks of MACEs in older individuals [3, 14, 15]. Given the influence of gender on risks also varies with age, gender is an essential demographic variable to be incorporated into a robust risk prediction model. The challenge in predicting MACEs in MHD patients stems from the intricate, non-linear nature of underlying risk factors, which necessitates more-complex risk stratification tools.

Machine learning (ML) algorithms offer a powerful alternative, capable of identifying complex patterns and non-linear relationships within high-dimensional clinical datasets without requiring pre-specified hypotheses about the nature of those interactions [16]. Therefore, in this study, we developed and validated an ML model to predict the risk of MACEs in patients on MHD. We hypothesized that a model incorporating both traditional cardiovascular risk factors and kidney disease-specific variables, such as mineral metabolism parameters, inflammatory markers, vascular calcification scores, and treatment-related profiles, would achieve superior predictive accuracy compared to traditional statistical approaches. By leveraging advanced feature selection and robust validation techniques, our goal was to create a clinically interpretable and practically viable risk prediction tool for use in MHD populations. Given the multifactorial nature of CVD risks in HD, identifying the best predictive features from this array was a challenge. Machine learning offers an opportunity to discern which combinations of clinical, laboratory, and echocardiographic parameters best predict MACE, enabling refined risk stratification and potentially earlier interventions.

## Methods

### Selection of patients

**Fig. 1:**
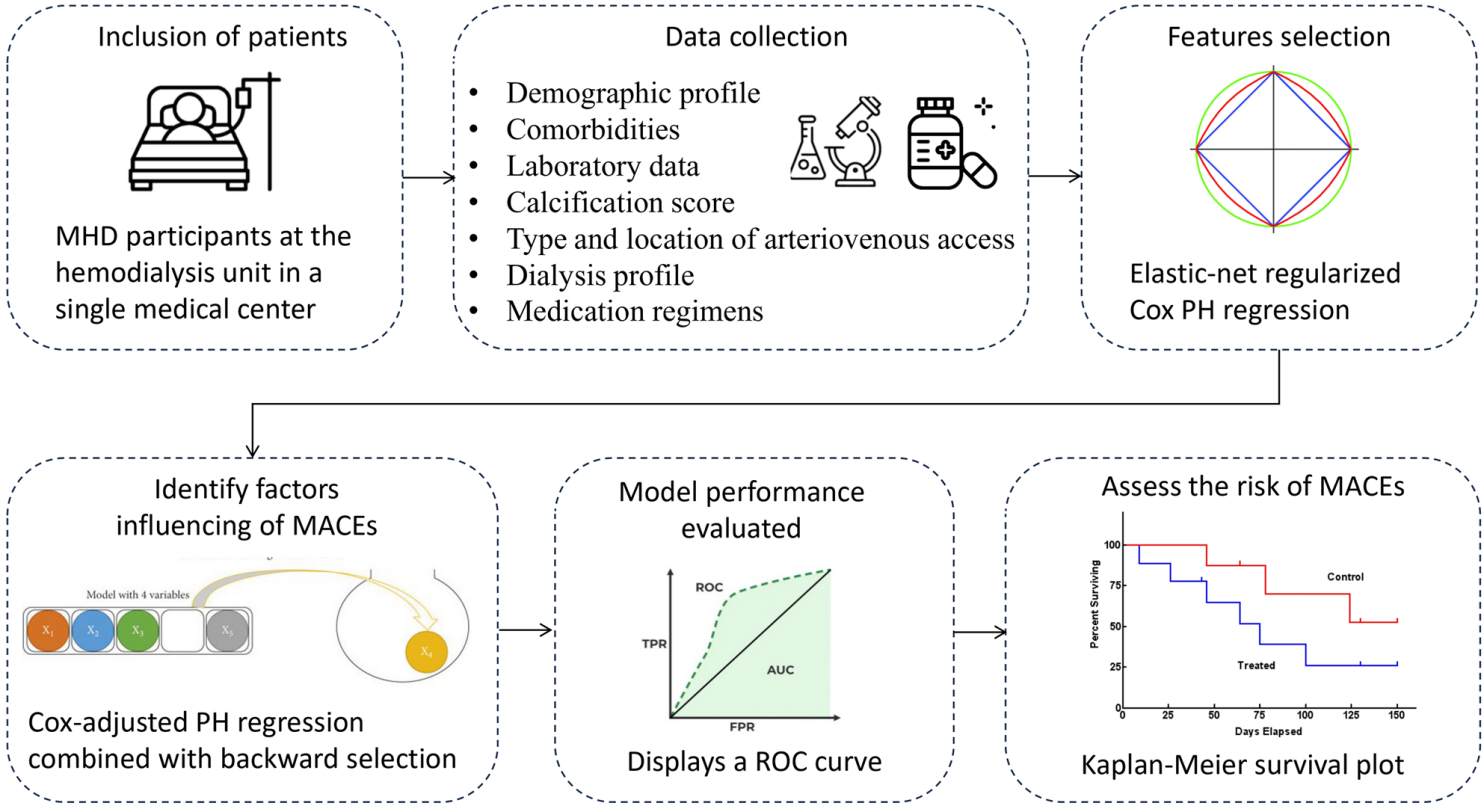
Methodological framework of the study

This cohort study included 412 adult MHD participants at the hemodialysis unit in a single medical center between October 1 and December 31, 2018. After participant identification, we collected their clinical features, including their demographic profile, comorbidities, laboratory parameters (serum biochemistry, complete blood count, and electrolyte panels), calcification score of the aortic arch (AoAC) on chest x-ray, blood pressure, type and location of arteriovenous access, dialysis profile including Kt/V (K is the dialyzer clearance of urea, t is dialysis time, and V is the volume of urea distribution), and medication regimens (antihypertensives, antidiabetics, statins, antiplatelets, and anticoagulants). In total, 412 individuals (192 [46.6%] female) with ESKD were included. These enrolled subjects were followed-up to death, modality or hemodialysis center transfer, or the end of the study on December 31, 2021. These enrolled adult MHD patients were ultimately divided into two groups based on the presence of MACEs. The flow diagram illustrating the selection of enrolled patients, including the inclusion and exclusion criteria, as shown in Figure S1. Additionally, we have created a schematic workflow to depict the overall methodology and the model development/validation pipeline, presented as Fig. 1.

### Outcome variables

The primary endpoint was the occurrence of a MACE, which was defined as any of the following events: hospitalization for myocardial infarction, coronary revascularization, stroke, or heart failure, or death from cardiovascular causes.

### Feature selection and risk model construction

To identify key predictors of MACEs in MHD patients, we implemented a systematic two-stage feature selection strategy combined with rigorous missing data handling procedures.

### Two-stage feature selection approach

Beginning with an initial pool of 85 candidate variables encompassing demographic characteristics, laboratory parameters, comorbidities, dialysis-related factors, and medication profiles, we employed a hybrid machine learning framework to systematically reduce dimensionality while preserving predictive performance.

In the first stage, we applied an elastic-net regularized Cox proportional hazards (PH) regression model using the *glmnet* package in R software. The elastic-net method was specifically selected for its dual capacity to manage multicollinearity through combined L1 (lasso) and L2 (ridge) penalties, making it particularly well-suited for high-dimensional clinical datasets with correlated predictors. The optimal regularization parameter (λ) was determined through 10-fold cross-validation, selecting the λ value that minimized the mean cross-validated partial likelihood deviance. This initial feature screening successfully identified a robust intermediate subset of 46 candidate predictors by shrinking coefficients of less informative variables toward zero while retaining those with the strongest predictive signals.

In the second stage, these 46 retained features were entered into a standard multivariate Cox PH regression model. We then implemented a backward stepwise selection procedure, iteratively removing variables based on statistical significance. Variables with p-values exceeding 0.05 were sequentially eliminated until all remaining predictors achieved statistical significance. This refinement process yielded a final parsimonious model comprising 13 variables, optimizing the balance between predictive accuracy, clinical interpretability, and model parsimony.

### Missing data imputation

Prior to model construction, we addressed missing data through a principled statistical framework to ensure the validity and reliability of our estimates. For the 13 variables included in the final Cox regression model, missing values were handled using multivariate imputation by chained equations (MICE), implemented under the missing at random (MAR) assumption. We generated 20 multiply imputed datasets, and parameter estimates along with their standard errors were pooled across these datasets according to Rubin’s rules. This approach appropriately accounted for both within-imputation and between-imputation variability, ensuring that uncertainty attributable to the imputation process was reflected in the final confidence intervals and hypothesis tests.

### Predictions of maces

To determine the optimal cutoff point for stratifying patients into high-risk and low-risk groups based on their predicted MACEs risk scores, the *survMisc* package in R was utilized for risk stratification. Kaplan-Meier survival curves were then constructed to illustrate the discrepancy in survival rates between the two groups. The log-rank test was employed to assess the statistical significance of survival differences.

### Model validation and performance evaluation

The performance of the final 13-variable prediction model was comprehensively assessed across three key domains: discrimination, calibration, and clinical utility, following established guidelines for clinical prediction model evaluation.

### Discrimination

Model discrimination, defined as the ability to distinguish between patients who would develop MACEs and those who would not, was quantified using the area under the receiver operating characteristic curve (AUROC). To obtain an unbiased estimate of discriminative accuracy and minimize overfitting bias, we employed leave-one-out (LOO) cross-validation. This rigorous internal validation approach iteratively trains the model on n-1 observations and tests it on the held-out observation, repeating this process for all patients to generate performance metrics that better reflect the model’s generalizability.

### Calibration

Model calibration, which evaluates the agreement between predicted probabilities and observed event rates, was assessed using both statistical and graphical methods. The Hosmer-Lemeshow goodness-of-fit test was performed to formally test calibration, where a non-significant p-value (*p* > 0.05) indicates adequate agreement between predicted and observed outcomes. To complement this statistical assessment, we generated a calibration plot using bootstrap resampling (1,000 iterations) to derive bias-corrected estimates. The calibration plot visually depicts the correspondence between predicted risk probabilities (x-axis) and observed event frequencies (y-axis), with the ideal calibration represented by a 45-degree line. Deviation from this ideal line indicates miscalibration, with points above the line suggesting underestimation and points below suggesting overestimation of risk.

### Clinical utility

The clinical utility of the prediction model was evaluated using Decision Curve Analysis (DCA), a method that quantifies the net benefit of using the model to guide clinical decision-making. DCA was performed across a range of risk threshold probabilities (0% to 100%), comparing the net benefit of model-guided decisions against two default strategies: treating all patients regardless of predicted risk (“treat all”) and treating no patients (“treat none”). The net benefit represents the difference between the true positive rate and the false positive rate, weighted by the odds of the threshold probability. A model demonstrating positive net benefit above the reference strategies across clinically relevant risk thresholds indicates practical value for clinical implementation.

### Statistical analysis

Baseline characteristics were analyzed using appropriate statistical tests based on the type and distribution of the data. Continuous variables were compared using independent *t*-tests for two groups, provided the data followed a normal distribution. For non-normally distributed continuous or ordinal data, the Kruskal-Wallis test was utilized as a non-parametric alternative. Categorical variables were analyzed using Chi-squared tests when expected cell frequencies were ≥ 5. Fisher’s exact test was applied for smaller sample sizes or sparse data in contingency tables. The proportional hazards assumption was formally tested using Schoenfeld residuals, with non-significant p-values (*p* > 0.05) for both the global model and individual predictors indicating that the assumption was satisfied. Results were reported as the mean (standard deviation (SD)) or number (%) for continuous variables and as proportions for categorical variables. Statistical significance was set to *p* < 0.05, and all analyses were performed using standard statistical software (R vers. 4.1.2) to ensure accuracy and reproducibility.

## Results

### Feature selection using elastic net regression

**Fig. 2:**
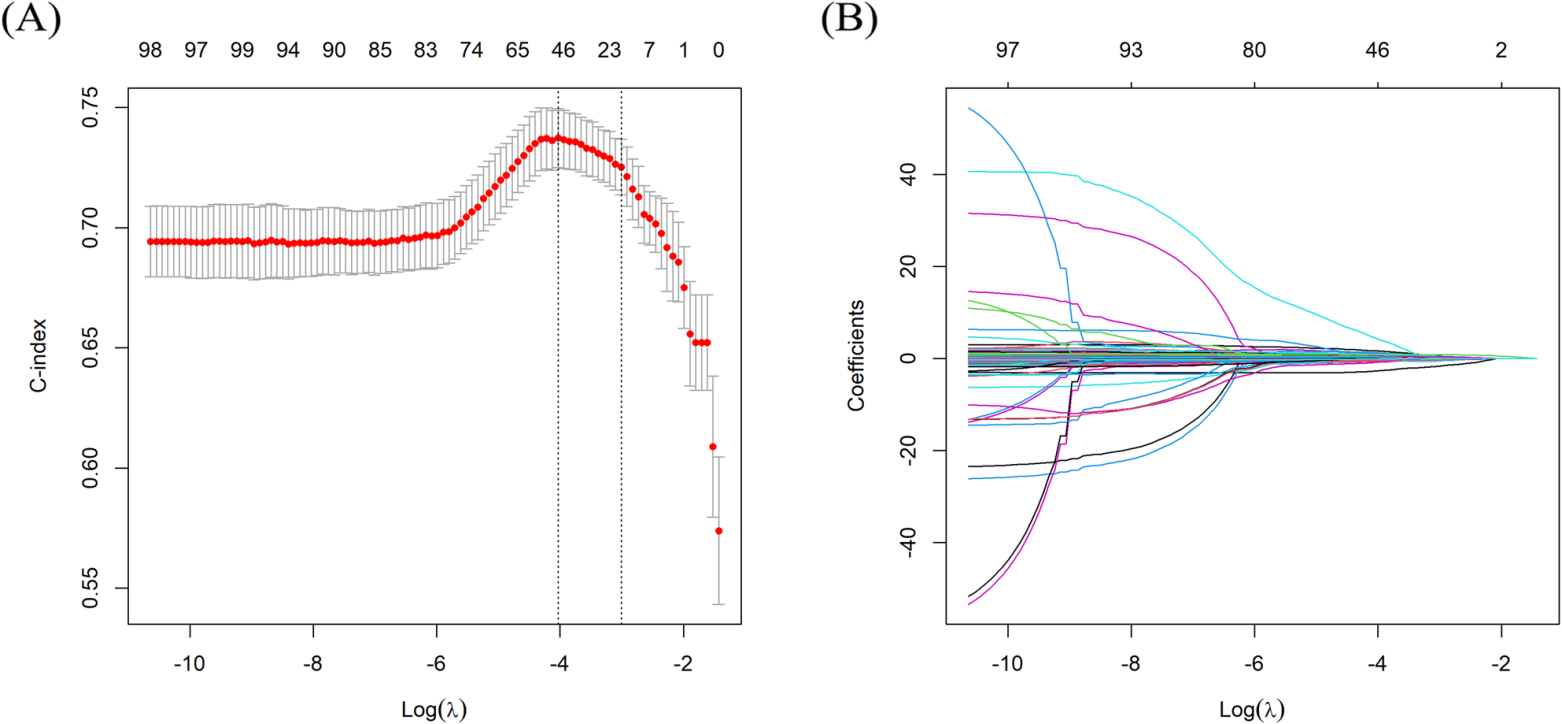
(**A**) Feature selection profile and (**B**) coefficient paths in the elastic-net regression

An elastic-net regression was employed for feature selection in a structured two-step process within the framework of the survival analysis. Here we applied an elastic-net regularized Cox PH regression, which integrates both ridge and lasso penalties, thus facilitating management of correlated predictors and enabling the selection of a robust set of variables from a high-dimensional dataset. Figure 2A illustrates the outcome of feature selection via the elastic-net regression, highlighting the optimal selection of 46 features. The x-axis represents the log of lambda, and the y-axis was used for a performance evaluation through the C-index. Results indicated that when feature selection was constrained to 46 features, the model achieved the highest discriminative power as measured by the C-index. This visualization demonstrates the efficacy of the selected features at the optimal lambda value in improving the model accuracy. Figure 2B illustrates coefficient trajectories for each predictor in the elastic-net model as a function of log(λ), thereby demonstrating how regularization influenced the selection of features.

In total, 412 individuals (192 [46.6%] female) with MHD were included. Table 1 summarizes differences in baseline characteristics between dialysis patients who experienced a MACE (*n* = 164) and those who did not (*n* = 248). Variables listed in Table 2 were selected using an elastic-net approach, emphasizing features most predictive of cardiovascular events. Table 1 reports key demographic and clinical data, including age, gender distribution, and a wide array of biochemical and hematological parameters. The results indicated a notable age discrepancy between the two groups, with MACE (+) patients exhibiting a higher average age than MACE (-) patients. A greater proportion of males was observed in the MACE (+) group compared to the MACE (-) group, although this difference was not statistically significant. Prevalences of diabetes mellitus, hypertension, coronary artery disease, and heart failure were significantly higher in the MACE (+) group. Furthermore, albumin and cholesterol levels were significantly lower in the MACE (+) group. This table presents a comprehensive comparison of baseline characteristics between dialysis patients with and those without MACEs. It highlights significant differences in age, gender, comorbidities, and biochemical markers. These findings, which were derived from variables selected via the elastic net, suggest important implications for patient management and risk stratification in a dialysis setting. Additional baseline characteristics that did not show statistically significant differences between the two groups are summarized in Supplementary Table 1 for completeness.

**Table 1:** Baseline characteristics of Hemodialysis patients with and those without a MACE

	MACE(-) (*n* = 248)		MACE(+) (*n* = 164)		*p* value
Age (years)	63.8	(12.7)	68.6	(11.5)	0.0001*
Gender					0.0572†
Male	123	(49.6)	97	(59.1)	
Female	125	(50.4)	67	(40.9)	
Comorbidity					
DM	102	(41.1)	96	(58.5)	0.0005†
Hypertension	191	(77.0)	141	(86.0)	0.0244†
Coronary artery disease	89	(35.9)	84	(51.2)	0.0020†
Acute myocardial infarction	3	(1.2)	9	(5.5)	0.0156‡
PAOD	52	(21.0)	59	(36.0)	0.0008†
Albumin (gm/dl)	3.9	(0.3)	3.8	(0.4)	0.0002*
Cholesterol (mg/dl)	160.1	(35.8)	150.4	(39.8)	0.0056*
Glucose [AC] (mg/dl)	111.7	(52.3)	120.3	(58.1)	0.0426*
HbA1c (g/dl)	10.5	(1.4)	10.2	(1.4)	0.0228*
CXR_AoAC					< 0.0001†
0: no calcification	91	(36.7)	29	(17.7)	
1: less than 50% calcification	51	(20.6)	32	(19.5)	
2: more than 50% calcification	51	(20.6)	56	(34.1)	
3: circumference calcification	35	(14.1)	37	(22.6)	
Heart rate	74.1	(12.6)	76.5	(12.6)	0.0276*
Antiplatelet	82	(33.1)	117	(71.3)	< 0.0001†
alpha-glucose inhibitor	1	(0.4)	10	(6.1)	0.0006‡
Amputation					0.0096‡
0: No	247	(99.6)	158	(96.3)	
1: Below knee	1	(0.4)	1	(0.6)	
2: After knee	0	(0.0)	5	(3.0)	
Insulin	32	(12.9)	53	(32.3)	< 0.0001†
Dialytic weight loss	2.4	(1.0)	2.7	(1.1)	0.0451*
RI/ACEI/ARB	120	(48.4)	97	(59.1)	0.0323†
Hb (g/dl)	10.5	(1.4)	10.2	(1.4)	0.0228*
TEF	63.8	(15.7)	59.3	(17.2)	0.0172*

Data are expressed as n (%) for categorical data and as mean ± standard deviation for continuous data. AC, ante cibum (before meals); CXR_AoAC, chest X-ray aortic arch calcification, graded on a 4-point scale (0 if no calcification, 1 if < 50% of the arch calcified, 2 if > 50% of the arch calcified, 3 if circumferential calcification); DM, diabetes mellitus; Hb, Hemoglobin; TEF, total ejection fraction; MACE, major adverse cardiovascular Event; PAOD, peripheral arterial occlusive disease; RI/ACEI/ARB, renin inhibitors, angiotensin-converting enzyme inhibitors and angiotensin II receptor blockers

*Kruskal-Wallis test †Chi-square test ‡Fisher’s exact test

**Table 2:** Feature selected by the elastic-net regression

No.	Features	Coef.	No.	Features	Coef.
1	Conductivity	4.417	26	Glucose[AC] (mg/dl)	-0.002
2	Age	1.653	27	Frequency	-0.004
3	Heart rate	1.279	28	Meglitinides	-0.076
4	Ca×P (mg^2^/dL^2^)	1.092	29	Diastolic dysfunction_mod	-0.085
5	Antiplatelet	0.920	30	Dialysis years	-0.087
6	Alpha-glucose inhibitor	0.870	31	Alpha-blocker	-0.099
7	Total protein (gm/dl)	0.778	32	Transferrin saturation (%)	-0.162
8	Amputation	0.615	33	Fe (ug/dl)	-0.230
9	AMI	0.605	34	Platelet (x1000/ul)	-0.336
10	Insulin	0.504	35	Hb (g/dl)	-0.350
11	Gender	0.445	36	AVA_left arm	-0.351
12	Fibrate	0.373	37	AVA_right forearm	-0.354
13	P (mg/dl)	0.355	38	CXR_AoAC_2	-0.369
14	Dialytic weight loss	0.297	39	Treatment time	-0.374
15	Anti-coagulants	0.258	40	TEF	-0.400
16	AVA_left forearm	0.250	41	R.B.C. (x10^6/ul)	-0.415
17	RI/ACEI/ARB	0.115	42	K (meq/l)	-0.472
18	CXR_AoAC_1	0.112	43	Kt/V (Gotch)	-0.522
19	HF	0.098	44	Cholesterol (mg/dl)	-0.607
20	Ferritin (ng/ml)	0.085	45	Weight	-0.869
21	A.S.T. [GOT] (IU/L)	0.084	46	Albumin (gm/dl)	-2.711
22	Hypotension during dialysis	0.069			
23	PTH (pg/ml)	0.057			
24	Diastolic dysfunction_mild	0.035			
25	Arrythmia	0.032			

AC, ante cibum (before meals); AMI, acute myocardial infarction; AST, aspartate aminotransferase; AVA, arteriovenous access; Ca×P, calcium-phosphate product; Coef., coefficient from the elastic-net regularized Cox model (positive values are associated with increased risk, negative values with decreased risk); CXR_AoAC, chest X-ray aortic arch calcification, graded on a 4-point scale (0 if no calcification, 1 if < 50% of the arch calcified, 2 if > 50% of the arch calcified, 3 if circumferential calcification); DM, diabetes mellitus; Hb, hemoglobin; HF, heart failure; TEF, total ejection fraction; Fe, iron; K, potassium; P, phosphorus; PTH, parathyroid hormone; RBC, red blood cell; RI/ACEI/ARB, renin inhibitors, angiotensin-converting enzyme inhibitors and angiotensin II receptor blockers

### Fitting a multivariate model to eliminate non-significant variables

To identify factors influencing MACE risks, we refined the feature set using a Cox-adjusted PH regression combined with backward selection. Backward selection is a stepwise regression method in which all variables are initially included, and subsequently, the least significant variables are removed in a sequential manner based on their statistical significance, as assessed by *p* values. The proportional hazards assumption was satisfied for the final 13-variable model, as evidenced by a non-significant global Schoenfeld residuals test (*p* = 0.528) and non-significant tests for all individual predictors (all *p* > 0.05). Table 3 presents a Cox regression analysis summarizing the association between the 13 selected features and the risk of MACEs. Each feature’s impact is expressed as a hazard ratio (HR) with a 95% confidence interval (CI). The final selection of features included gender, location of an arteriovenous access (AVA), treatment frequency, AoAC on chest x-ray, albumin, cholesterol, hemoglobin (Hb), platelets, the calcium-phosphate product (Ca×P), weight, insulin use, antiplatelet use, and anticoagulant use.

**Table 3:** Cox regression analysis of 13 selected features for maces

	Full Model	
	HR (95%CI)	*p* or *p* for trend
Gender		0.0138
Female	1.00 (ref.)	
Male	2.19 (1.17–4.07)	
Location of AVA		0.0020
Left forearm	1.00 (ref.)	
Left arm	0.10 (0.03–0.35)	
Right forearm	0.48 (0.17–1.34)	
Right arm	0.78 (0.28–2.17)	
Treatment frequency		0.4136
TIW	1.00 (ref.)	
BIW	1.49 (0.57–3.87)	
CXR_AoAC		0.0114
no calcification	1.00 (ref.)	
less than 50% calcification	0.95 (0.41–2.21)	
more than 50% calcification	2.43 (1.25–4.72)	
circumference calcification	2.32 (0.93–5.78)	
Albumin	0.30 (0.12–0.76)	0.0110
Cholesterol	0.99 (0.98-1.00)	0.0536
Hb	0.79 (0.64–0.98)	0.0295
Platelet	0.99 (0.99-1.00)	0.0488
Ca×P	1.03 (1.01–1.05)	0.0063
Weight	0.97 (0.95-1.00)	0.0253
Insulin		< 0.0001
No	1.00 (ref.)	
Yes	4.04 (2.09–7.82)	
Antiplatelet		0.0005
No	1.00 (ref.)	
Yes	2.75 (1.56–4.87)	
Anti-coagulants		0.0070
No	1.00 (ref.)	
Yes	6.39 (1.66–24.6)	

AVA, arteriovenous access; BIW, bis in die (twice a week) hemodialysis treatment schedule; TIW, ter in die (three times a week) hemodialysis treatment schedule; Ca×P, calcium-phosphate product; CI, confidence interval; CXR_AoAC, chest X-ray aortic arch calcification, graded on a 4-point scale (0 if no calcification, 1 if < 50% of the arch calcified, 2 if > 50% of the arch calcified, 3 if circumferential calcification); Hb, hemoglobin; HR, hazard ratio; MACE, major adverse cardiovascular event; ref, reference group

The analytical results showed that a male gender was associated with over twice the hazard risk (HR = 2.19) compared to the female reference group. The location of an AVA exhibited varying risks, with the left upper arm showing a notably reduced hazard (HR = 0.10) compared to the reference. Treatment frequency did not show a significant trend. Higher AoAC on chest x-ray scores were associated with an increased hazard, particularly for a score of 2. Higher albumin levels were linked with a lower risk of MACEs (HR = 0.30). A one-unit increase in Hb and platelet counts slightly decreased the hazard, while an increase in the Ca×P raised the risk. Body weight had a protective effect (HR = 0.97 per unit increase). Insulin usage was associated with a fourfold increased hazard. Use of antiplatelets and anticoagulants were also associated with significantly increased hazards (HR = 2.75 and 6.39, respectively). These results highlight the multifactorial nature of MACE risks, where each feature contributed differently to the overall hazard.

### Predictive model of maces in MHD patients

**Fig. 3:**
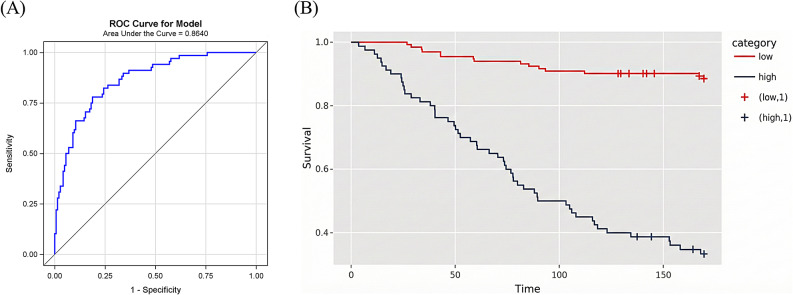
(**A**) ROC curve for a MACE predictive model based on an elastic net regression (**B**) Kaplan-Meier survival plot between patients in the high-risk (black line) and low-risk (red line) groups for MACEs

Figure 3A displays a receiver operating characteristic (ROC) curve for a predictive model based on an elastic-net regression that calculated risk scores for each sample. The model’s performance was evaluated using the LOO cross-validation method. The ROC curve is a plot of sensitivity (true positive rate) versus [1 – specificity] (false positive rate) at various threshold settings. The AUROC value of 0.864 (CI: 0.8131–0.9148) indicated a high level of accuracy and reliability for the model’s ability to distinguish between outcomes. Figure 3B depicts the Kaplan-Meier survival plot, which was derived from the elastic-net regression model that utilized 13 features to assess the risk of MACEs in MHD patients. The figure illustrates the existence of two distinct groups: a high-risk group (depicted by the black line) and a low-risk group (depicted by the red line). The groups were determined based on the cut-point (0.3263) for MACE risk, identified using the survMisc R package. The plot shows a marked difference in survival probability over time, with the high-risk group experiencing a lower survival rate compared to the low-risk group. A notable discrepancy in incidences of MACEs between the two groups was evident (log-rank *p* value < 0.001).

### Model calibration and clinical utility

**Fig. 4:**
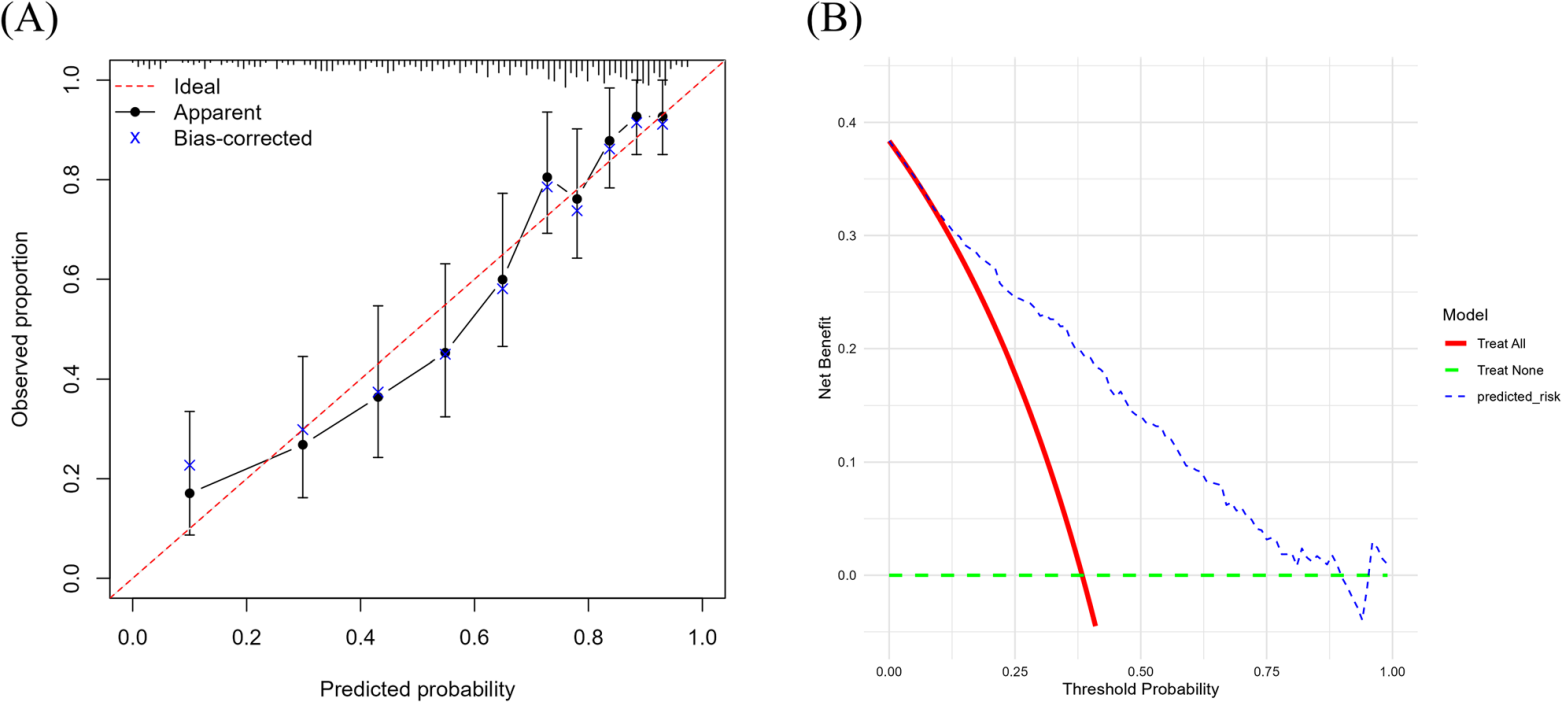
(**A**) Calibration plot for MACE. The x-axis represents the predicted probability of MACEs, and the y-axis represents the observed proportion of MACEs. The dashed red line (“Ideal”) indicates perfect calibration. The solid black line (“Apparent”) shows the model’s performance on the development dataset, while the blue ‘x’ marks (“Bias-corrected”) represent the optimism-corrected calibration derived from bootstrap validation, which closely follow the ideal line. (**B**) Decision Curve Analysis for MACE. The y-axis represents the net benefit, and the x-axis represents the threshold probability. The blue dashed line (“predicted_risk”) shows the net benefit of the 13-variable model. The model provides a greater net benefit than the default strategies of “Treat All” (red solid line) or “Treat None” (green dashed line) across a wide range of clinically relevant risk thresholds (approximately 10% to 75%)

The final 13-variable model exhibited good calibration performance. The Hosmer-Lemeshow goodness-of-fit test yielded a non-significant result (*p* = 0.591), indicating adequate agreement between predicted probabilities and observed event rates. Calibration was further confirmed visually through the calibration plot (Fig. 4A), in which the bias-corrected estimates derived from bootstrap validation closely approximated the 45-degree line representing ideal calibration. The observed deciles of predicted risk aligned well with the diagonal reference line, demonstrating that the model provides accurate risk estimates across the spectrum of predicted probabilities.

Decision Curve Analysis (DCA) demonstrated the clinical utility of the prediction model (Fig. 4B). Across a wide range of clinically relevant risk thresholds (10% to 75%), the 13-variable model yielded greater net benefit compared to the default strategies of treating all patients or treating no patients. This finding indicates that model-guided risk stratification has practical value for clinical decision-making and could facilitate more efficient allocation of preventive interventions in maintenance hemodialysis patients.

## Discussion

The current study was conducted to develop and validate an explainable ML model for MACE risk predictions among MHD patients. The model incorporates 13 input features which demonstrated the best performance among the 85 clinical variables examined. Among the input features, the use of antiplatelets and anticoagulants are two well-known features that have been widely used in various diagnostic and prognostic models in patients with MHD of various etiologies [17–20]. Stenosis or thrombosis of dialysis fistulas over the years is an inevitable result secondary to repeated punctures and the traumatizing effect of arterialized rapid flow on the venous wall. Most vascular access complications are attributed to stenosis and thromboses and ultimately require interventions in order to maintain scheduled HD. Antiplatelet therapy is frequently initiated in high-risk individuals but may also reflect a preexisting atherosclerotic burden, which inherently elevates MACE risks. On the other hand, patients who use heparin anticoagulation will occasionally develop an immune-mediated adverse drug reaction of heparin-induced thrombocytopenia (HIT). HIT patients develop hypercoagulability with greatly increased risk of thromboses, both venous and arterial.

The Ca×P parameter was also previously reported to be a robust predictor of survival in patients with CKD [21, 22]. Hyperphosphatemia is an important problem in hemodialysis patients. Elevated levels of phosphate and Ca×P are associated with extraskeletal calcifications, as well as increased vascular calcification. Progression of vascular calcification was strongly correlated with cardiovascular events. Cardiovascular calcifications may possibly be related to the high cardiovascular mortality seen in dialysis patients. Dysregulated mineral metabolism such as a calcium-phosphate imbalance is a critical driver. These findings underscore the need for multifactorial risk assessments in this population.

Low serum albumin level, high aortic arch calcification, and abnormal high Ca×P product warrant aggressive management of nutrition, vascular disease, and mineral metabolism. Managements include nutritional support through enteral or intradialytic parenteral nutrition, prevention of atherosclerosis, and stricter phosphate control. Identifying hemodialysis patients receiving antiplatelet or anticoagulant therapy assists in balancing the risks of thrombosis and bleeding [23]. For insulin users, close monitoring of glucose levels and prevention of hypoglycemia should be prioritized. Low hemoglobin and cholesterol level may indicate the need for early anemia treatment and nutritional assessment to reduce risk of cardiovascular disease in the population [24]. Risk models incorporating these features enable clinicians to identify individuals who require closer surveillance, multidisciplinary care, or early intervention [25].

The elastic net combines L1 (LASSO) and L2 (ridge) regularization, effectively handling correlated predictors common in CKD datasets. This dual penalty reduces overfitting in prediction models, improving the root mean squared error (RMSE) compared to a standard regression. In stroke risk predictions for revascularized coronary artery disease (CAD) patients, the elastic net helped reduce 35 variables to 14 clinically relevant features, while maintaining an AUROC of 0.85. Similarly, it identified critical MACE predictors like the right ventricular ejection fraction (AUROC = 0.82) in congenital heart disease cohorts.

The stepwise selection method is the most widely used method of variable selection. Stepwise methods automated variable selection from 46 candidate predictors, reducing the computational complexity, while identifying statistically significant variables linked to MACEs. This aligns with clinical needs for parsimonious models deployable in resource-limited settings. Retained variables provide clinically actionable insights through hazard ratios, maintaining transparency compared to “black-box” ML models. For instances in Table 3, our study indicated that 2.19 times as many male patients were experiencing MACEs compared to female patients in CKD cohorts. Stepwise selection is familiar to clinicians, thereby facilitating its adoption.

The hybrid approaches in this study which used an elastic net for initial feature screening, then applied interpretable ML for final modeling, providing a comprehensive framework for personalized medicine in CKD management. From a practical standpoint, integrating these variables into a clinical decision support framework may enhance risk stratification at key time points. Additionally, integrating variables for risk stratification involves combining diverse data points to categorize patients into risk groups, which allow for tailored interventions [1, 26]. This approach extends beyond traditional, single-variable methods by using a broader range of data to enhance the accuracy of risk prediction. Gender and the location of arteriovenous access influence the depth and the diameter of vascular access, which are related to dialysis adequacy. Moreover, treatment frequency can affect toxin clearance and volume status, ultimately impacting cardiovascular outcomes. The Ca×P product is associated with vascular calcification and cardiovascular mortality in hemodialysis patients. Aortic arch calcification has been shown to correlate positively with coronary artery disease and can therefore be regarded as an independent predictor of MACE. Body weight reflects nutritional status and fluid balance, factors that may collectively influence cardiovascular outcomes. Low serum albumin levels may indicate inflammation and malnutrition, whereas cholesterol, hemoglobin, and platelet levels are linked to atherosclerosis, anemia, and bleeding or thrombotic risks—all highly prognostic for cardiovascular outcomes in dialysis patients. Prescriptions for insulin, antiplatelet, and anticoagulant agents serve as surrogate markers for underlying diabetes, thrombotic risk, and pre-existing comorbidities. Their use may help identify higher-risk patient groups or therapy-related complications. Understanding the interplay among these variables enables health systems to allocate resources more effectively and to implement targeted care strategies across different risk strata [27]. A tool based on the current model could assist clinicians in identifying patients who warrant more-intensive cardiology surveillance. For example, even in the absence of overt symptoms, tailored cardioprotective interventions may provide benefits. Our model demonstrated competitive accuracy within an CKD-specific context, but its value lies in interpretability and domain alignment rather than in numerical superiority alone. The model has potential as a clinically meaningful adjunct to existing practice other than merely being a replacement.

This study has several important limitations. First, the modest events-per-variable (EPV) ratio presents challenges for model reliability. With 164 MACE events and 13 predictor variables (EPV ≈ 12.6), our model marginally exceeds the “one-in-ten rule” guideline, which recommends at least 10 events per predictor to ensure stable coefficient estimates and minimize overfitting. This modest EPV ratio, combined with feature selection from 85 initial candidates, increases several methodological concerns. The model may capture spurious associations that do not replicate in external datasets, coefficient estimates may exhibit reduced precision with wider confidence intervals, and the risk of type I errors is elevated. When EPV ratios fall below 15–20, models become more susceptible to overfitting, meaning their apparent performance in the development cohort may not generalize well to new patients.

To mitigate these risks, we employed a rigorous two-stage feature selection strategy combining elastic-net regularization (which penalizes model complexity) with backward stepwise selection, and conducted comprehensive internal validation including leave-one-out cross-validation, calibration assessment (Hosmer-Lemeshow *p* = 0.591), and decision curve analysis. These approaches provided converging evidence of model stability and clinical utility. However, internal validation cannot fully address overfitting concerns or confirm generalizability.

Second, the single-center retrospective design limits generalizability. Our cohort from a single Taiwanese hemodialysis center may have distinct characteristics regarding patient demographics, comorbidity patterns, dialysis practices, vascular access management, and healthcare system factors that differ from other populations. Center-specific practices in managing mineral bone disorder, anticoagulation strategies, and cardiovascular risk may influence both baseline risk profiles and predictor importance. The retrospective design also introduces potential selection and information biases.

Given the modest EPV ratio and single-center design, external validation in independent, multicenter cohorts is essential before clinical implementation. Such validation should evaluate whether the identified predictors retain prognostic value across diverse settings, whether model calibration remains adequate in populations with different risk profiles, and whether clinical utility translates to real-world benefit. Until external validation is completed, our model should be considered hypothesis-generating rather than ready for routine clinical application. Moreover, limited data restrict the ability to detect or model non-linear relationships and interactions between variables. In the field of clinical predictions, complex pathophysiological relationships such as the joint effects of mineral metabolism disturbances and inflammation on cardiovascular risks in dialysis patients might thus be overlooked. Finally, the small sample size also reduced the statistical power, increasing the risk that the study failed to detect true but modest associations, and precluded more-granular subgroup analyses (e.g., by age, sex, or comorbidity burden). Collectively, these limitations underscore the need for validation in larger, prospective, and ideally multicenter cohorts to confirm the reliability and generalizability of the proposed prediction model. They also support a careful, a priori approach to variable selection, favoring clinical plausibility and parsimony over maximizing statistical significance in small datasets.

In conclusion, this study developed a 13-variable prognostic model for predicting MACEs in maintenance hemodialysis patients using a two-step hybrid machine learning approach. By combining an elastic-net regression and Cox proportional hazards models with backward selection, the model provides a clinically interpretable tool that integrated traditional and non-traditional risk factors with key predictors included markers of MIA syndrome, CKD-MBD, and proxies for advanced comorbidities (e.g., medications used). However, given the retrospective, single-center study design, these findings should be considered preliminary, and further validation in larger prospective multicenter studies is required before considering its potential for clinical application.

## Supplementary Information

Below is the link to the electronic supplementary material.

Supplementary Material 1

Supplementary Material 2

## Data Availability

The raw data for conducting this analysis are available upon reasonable request to the corresponding author.

## Abbreviations

CVDs: Cardiovascular diseases
ESKD: End-stage kidney disease
MACEs: Major adverse cardiovascular events
CKD: Chronic kidney disease
MHD: Chronic hemodialysis
AUROC: Area under the receiver operating characteristic curve
AoAC: Aortic arch calcification
MIA: Malnutrition-inflammation-atherosclerosis
ML: Machine learning
Kt/V: Dialysis adequacy
PH: Proportional hazard
LOO: Leave-one-out
SD: Standard deviation
CI: Confidence interval
HR: Hazard ratio
AVA: Arteriovenous access
Hb: Hemoglobin
CXR: Chest x-ray
ROC: Receiver operating characteristic
AC: Ante cibum (before meals)
BMD: Mineral and bone metabolism disorder
QW: Quaque week (once a week)
BIW: Bis in die (twice a week)
TIW: Ter in die (three times a week)
Hct: hematocrit
RMSE: Root mean square error
HIT: Heparin-induced thrombocytopenia
CAD: Coronary artery disease
PAOD: Peripheral arterial occlusive disease
Ca×P: Calcium-phosphate product
HF: Heart failure
PTH: Parathyroid hormone
RBC: Red blood cell
WBC: White blood cell
AST: Aspartate aminotransferase
ALT: Alanine aminotransferase
TIBC: Total iron-binding capacity
TEF: Total ejection fraction
AMI: Acute myocardial infarction
RI/ACEI/ARB: Renin-angiotensin system inhibitors
DM: Diabetes mellitus
MCV: Mean corpuscular volume
