# Supplementary figures and images for: Exploring prognostic factors on vascular outcomes among maintenance dialysis patients and establishing a prognosis prediction model using machine learning methods
Source: BMC Med Inform Decis Mak. 2025 Dec 5;26:6. doi: 10.1186/s12911-025-03302-2 (PMC12797654; doi:10.1186/s12911-025-03302-2)

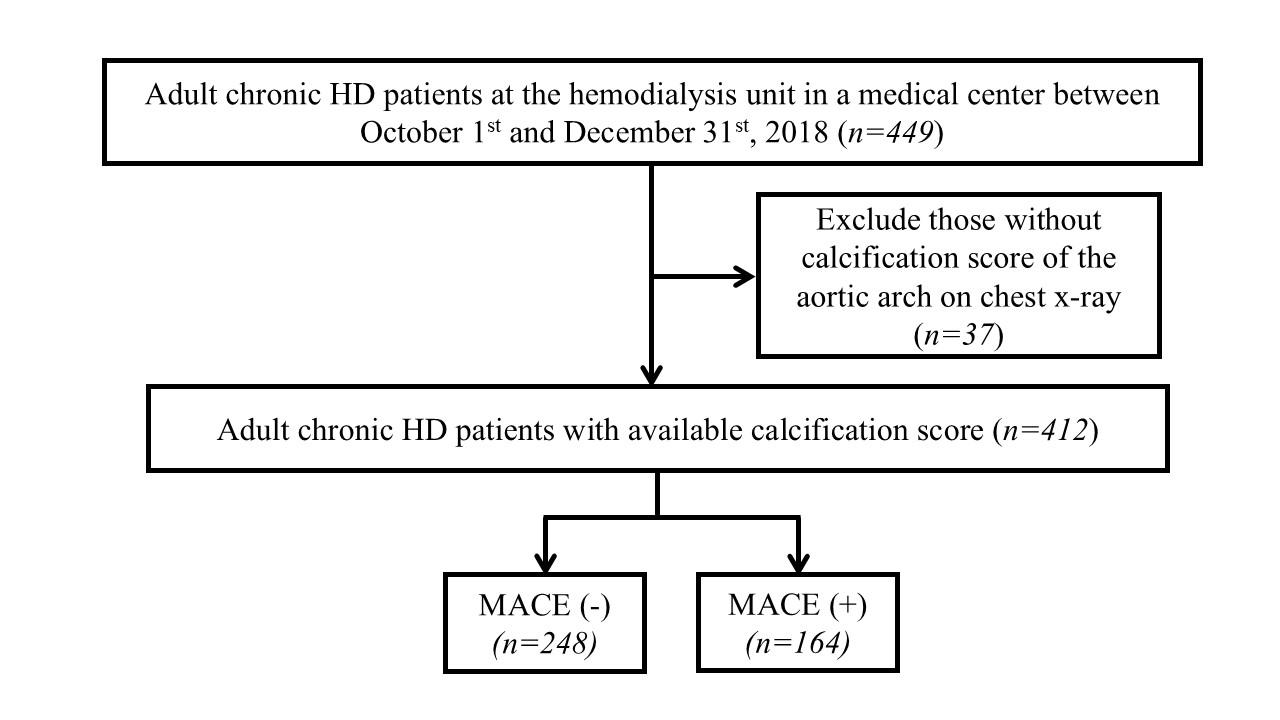

Supplement: Supplementary file 1 — Supplementary Material 1 [file 12911_2025_3302_MOESM1_ESM.jpg]
