# Supplementary material for: Exploring prognostic factors on vascular outcomes among maintenance dialysis patients and establishing a prognosis prediction model using machine learning methods
Source: BMC Med Inform Decis Mak. 2025 Dec 5;26:6. doi: 10.1186/s12911-025-03302-2 (PMC12797654; doi:10.1186/s12911-025-03302-2)
Supplement: Supplementary file 2 — Supplementary Material 2 [file 12911_2025_3302_MOESM2_ESM.docx]

**Supplementary Table 1**. Baseline characteristics of hemodialysis patients with and those without a MACE

|  | MACE(-) (n=248) | | MACE(+) (n=164) | | *p* value |
| --- | --- | --- | --- | --- | --- |
| Height (cm) | 161.8 | (8.9) | 161.7 | (7.9) | 0.9418* |
| Weight (kg) | 60.2 | (13.4) | 59.8 | (13.0) | 0.9111* |
| Systolic BP (mmHg) | 142.3 | (25.0) | 146.4 | (24.9) | 0.1182* |
| Diastolic BP (mmHg) | 68.8 | (13.8) | 66.4 | (16.2) | 0.0610* |
| Hyperlipidemia | 131 | (52.8) | 89 | (54.3) | 0.7734† |
| Cerebrovascular accident | 4 | (1.6) | 6 | (3.7) | 0.2057‡ |
| Heart failure | 43 | (17.3) | 48 | (29.3) | 0.0043† |
| COPD | 22 | (8.9) | 16 | (9.8) | 0.7612† |
| Malignancy | 27 | (10.9) | 14 | (8.5) | 0.4353† |
| Total protein (gm/dl) | 6.8 | (0.5) | 6.8 | (0.6) | 0.6013* |
| A.S.T. [GOT] (IU/L) | 16.1 | (7.0) | 17.1 | (13.6) | 0.9581* |
| A.L.T. [GPT] (IU/L) | 12.4 | (7.1) | 13.2 | (16.6) | 0.6035* |
| Alkaline-P (IU/L) | 73.6 | (39.9) | 80.7 | (41.6) | 0.0325* |
| Total Bilirubin (mg/dl) | 0.5 | (0.2) | 0.6 | (0.2) | 0.6290* |
| Triglyceride (mg/dl) | 149.1 | (119.1) | 133.9 | (96.1) | 0.1878* |
| W.B.C. (x1000/ul) | 6.9 | (2.0) | 6.9 | (1.8) | 0.6259* |
| R.B.C. (x10^6/ul) | 3.4 | (0.5) | 3.3 | (0.5) | 0.1353* |
| Hct (%) | 31.4 | (4.3) | 30.6 | (4.2) | 0.0763* |
| MCV (fl) | 94.2 | (7.8) | 94.2 | (7.0) | 0.5333* |
| Platelet (x1000/ul) | 195.9 | (55.8) | 187.7 | (63.3) | 0.1143* |
| Fe (ug/dl) | 76.9 | (32.3) | 71.9 | (34.6) | 0.0898* |
| TIBC (ug/dl) | 241.9 | (46.6) | 240.9 | (52.0) | 0.7962* |
| Ferritin (ng/ml) | 535.8 | (248.0) | 544.2 | (320.3) | 0.5178* |
| Transferrin saturation (%) | 32.3 | (13.2) | 29.9 | (12.6) | 0.0746* |
| Al (ng/ml) | 6.9 | (3.9) | 6.9 | (4.0) | 0.7292* |
| Weight after dialysis (kg) | 60.2 | (13.5) | 60.3 | (13.0) | 0.7538* |
| Uric acid (mg/dl) | 6.3 | (1.6) | 6.3 | (1.6) | 0.4672* |
| Na (meq/l) | 138.2 | (2.9) | 137.9 | (3.1) | 0.2165* |
| K (meq/l) | 4.7 | (0.6) | 4.6 | (0.7) | 0.1498* |
| Calcium (mg/dl) | 4.6 | (0.5) | 4.6 | (0.5) | 0.5748* |
| P (mg/dl) | 5.0 | (1.3) | 5.3 | (1.5) | 0.1052* |
| Kt/V | 1.4 | (0.2) | 1.4 | (0.2) | 0.1182* |
| PTH (pg/ml) | 287.9 | (273.8) | 344.2 | (364.2) | 0.3627* |
| Ca×P (mg^2^/dL^2^) | 45.9 | (12.8) | 48.5 | (13.6) | 0.0815* |
| Location of AVA |  |  |  |  | 0.5240† |
| Left forearm | 181 | (73.0) | 125 | (76.2) |  |
| Left arm | 38 | (15.3) | 18 | (11.0) |  |
| Right forearm | 21 | (8.5) | 13 | (7.9) |  |
| Right arm | 8 | (3.2) | 8 | (4.9) |  |
| Diastolic dysfunction |  |  |  |  | 0.4741‡ |
| No detection | 56 | (22.6) | 46 | (28.0) |  |
| None: 0 | 113 | (45.6) | 74 | (45.1) |  |
| Mild: 1 | 69 | (27.8) | 37 | (22.6) |  |
| Moderate: 2 | 8 | (3.2) | 7 | (4.3) |  |
| Severe: 3 | 2 | (0.8) | 0 | (0.0) |  |
| Conductivity | 14.0 | (0.3) | 14.0 | (0.1) | 0.3073* |
| Fibrate | 5 | (2.0) | 6 | (3.7) | 0.3577‡ |
| Anti-coagulants | 8 | (3.2) | 12 | (7.3) | 0.0586† |
| Hypotension during dialysis | 64 | (25.8) | 31 | (18.9) | 0.9684† |
| Arrythmia | 24 | (9.7) | 22 | (13.4) | 0.2384† |
| Frequency |  |  |  |  | 0.7669‡ |
| QW | 1 | (0.4) | 1 | (0.6) |  |
| BIW | 16 | (6.5) | 9 | (5.5) |  |
| TIW | 145 | (58.5) | 69 | (42.1) |  |
| Meglitinides | 20 | (8.1) | 19 | (11.6) | 0.2321† |
| Dialysis year | 9.1 | (7.8) | 7.4 | (6.1) | 0.1626* |
| Alpha-blocker | 30 | (12.1) | 16 | (9.8) | 0.4602† |
| Treatment time | 3.8 | (0.3) | 3.9 | (0.3) | 0.0686* |
| Data are expressed as n (%) for categorical data and as mean ± standard deviation for continuous data. AC, ante cibum (before meals); BP, blood pressure; QW, quaque week (once a week); BIW, bis in die (twice a week) hemodialysis treatment schedule; TIW, ter in die (three times a week) hemodialysis treatment schedule; AST, aspartate aminotransferase; ALT, alanine aminotransferase; Hct, hematocrit; TIBC, total iron-binding capacity; MCV, mean corpuscular volume; AVA, arteriovenous access; Ca×P, calcium-phosphate product; TEF, total ejection fraction; Fe, iron; K, potassium; P, phosphorus; Al, aluminum; PTH, parathyroid hormone; RBC, red blood cell; WBC, white blood cell; Kt/V, dialysis adequacy; RI/ACEI/ARB, renin-angiotensin system inhibitors, angiotensin-converting enzyme inhibitors and angiotensin II receptor blockers. | | | | | |
| *Kruskal-Wallis test. †Chi-square test. ‡Fisher’s exact test. | | | | | |
